# IFNGR1 signaling is associated with adverse pregnancy outcomes during infection with malaria parasites

**DOI:** 10.1371/journal.pone.0185392

**Published:** 2017-11-08

**Authors:** Mamoru Niikura, Shin–Ichi Inoue, Shoichiro Mineo, Hiroko Asahi, Fumie Kobayashi

**Affiliations:** 1 Department of Infectious Diseases, Kyorin University School of Medicine, Tokyo, Japan; 2 Department of Molecular Pathology, Tokyo Medical University, Tokyo, Japan; Ehime Daigaku, JAPAN

## Abstract

Complicated/severe cases of placental pathology due to *Plasmodium falciparum* and *P*. *vivax*, especially adverse pregnancy outcomes during *P*. *vivax* infection, have been increasing in recent years. However, the pathogenesis of placental pathology during severe malaria is poorly understood, while responses against IFN-γ are thought to be associated with adverse pregnancy outcomes. In the present study, we explored the role of IFN-γ receptor 1 (IFNGR1) signaling in placental pathology during severe malaria using luciferase-expressing rodent malaria parasites, *P*. *berghei* NK65 (*Pb*NK65L). We detected luciferase activities in the lung, spleen, adipose tissue, and placenta in pregnant mice, suggesting that infected erythrocytes could accumulate in various organs during infection. Importantly, we found that fetal mortality in IFNGR1-deficient mice infected with *Pb*NK65L parasites was much less than in infected wild type (WT) mice. Placental pathology was also improved in IFNGR1-deficient mice. In contrast, bioluminescence imaging showed that parasite accumulation in the placentas of IFNGR1-deficient pregnant mice was comparable to that in WT mice infected with *Pb*NK65L. These findings suggest that IFNGR1 signaling plays a pivotal role in placental pathology and subsequent adverse pregnancy outcomes during severe malaria. Our findings may increase our understanding of how disease aggravation occurs during malaria during pregnancy.

## Introduction

Malaria is a devastating parasitic disease in tropical and subtropical regions, with an estimated 214 million cases and 438,000 deaths per year [1]. The populations at greatest risk of developing severe pathology are children under the age of 5 years and pregnant women in areas where *Plasmodium falciparum* is endemic. Placental malaria is characterized by the accumulation of infected erythrocytes and inflammatory cells in the placenta [2, 3]. Placental malaria has been reported to be correlated with adverse pregnancy outcomes such as fetal growth restriction, still birth, premature delivery and, possibly, preeclampsia [4, 5].

*Plasmodium falciparum* erythrocyte membrane protein 1 (PfEMP1) is encoded by the *var* gene family of *P*. *falciparum* and expressed on membranes of infected erythrocytes. PfEMP1 has been involved in the adhesion of infected erythrocytes to endothelial cells by interacting with several molecules, such as CD36 and intercellular adhesion molecule-1 (ICAM-1) [6]. Switching of *var* gene expression alters the adhesion and antigenic phenotype of the infected erythrocyte. VAR2CSA is a variant of the PfEMP1 family of adhesion antigens and VAR2CSA-expressing erythrocytes infected with *P*. *falciparum* are detected in the placenta [7]. VAR2CSA is associated with sequestration in the placenta by binding to chondroitin sulfate A (CSA), which is expressed by the placental syncytiotrophoblast layer [8–11]. VAR2CSA recombinant domains are recognized by IgG from residents in endemic areas in a gender specific and parity-dependent manner [12].

Placentas from pregnant women infected with *P*. *falciparum* show infiltration of inflammatory cells such as monocytes, macrophages, and neutrophils [13, 14], and the production of cytokines such as gamma interferon (IFN-γ) and tumor necrosis factor (TNF) [15–19], suggesting that these inflammatory responses cause adverse effects, such as placental pathology during pregnancy.

*Plasmodium vivax* malaria has been considered a benign infection. However, there has been an increase in the reported cases of severe malaria due to *P*. *vivax* in recent years. Moreover, several studies reported that *P*. *vivax* infections were associated with placental malaria [13] and adverse pregnancy outcomes [20–24]. A multigene family orthologous to the *P*. *falciparum var* genes is not found in the genome of *P*. *vivax*. However, *P*. *vivax*-infected erythrocytes have been shown to adhere to placental cryosections and CSA [25, 26], although their cytoadhesion levels are 10-times lower than those of *P*. *falciparum*-infected erythrocytes. The adhesion of *P*. *vivax*-infected erythrocytes may be in part mediated by VIR proteins, encoded by *P*. *vivax* variant genes (*vir*) [26]. However, the pathogenesis of the placental pathology in severe vivax malaria during pregnancy is not completely understood, although IFN-γ and TNF-α receptor signaling in immune cells is predicted to be associated with adverse pregnancy outcomes.

The rodent malaria parasites *P*. *berghei* and *P*. *chabaudi* are useful for exploring the mechanism by which adverse pregnancy outcomes occur during severe malaria [27–31]. Rodent malaria parasites lack the ortholog to the *P*. *falciparum var* gene family in their genomes, but *P*. *berghei*-infected erythrocytes can adhere to placental tissue by binding to CSA [30]. Studies using *P*. *berghei* NK65 or *P*. *chabaudi* have suggested that MyD88 [30], IFN-γ and TNF [31] are associated with adverse effects during pregnancy. However, it remains unclear whether IFN-γ receptor signaling in cells and/or fetal cells is associated with placental pathology. In this study, we examined the role of IFN-γ receptor signaling in the pathogenesis of placental pathology during infection with *P*. *berghei* NK65 using IFN-γ receptor 1-deficient mice. We also examined the organs in which *P*. *berghei* NK65-infected erythrocytes accumulate in IFN-γ receptor 1-deficient mice using bioluminescence imaging, including the lung, liver, spleen, adipose tissues, and placenta.

## Materials and methods

### Animals and mating

Female and male C57BL/6J (B6) mice (5–6 weeks old) were purchased from CLEA Japan Inc. (Tokyo, Japan). IFN-γ R1-deficient mice (which lack the receptor for IFN-γ [32]) were purchased from Jackson Laboratories (Bar Harbor, MNE, USA). The experiments were approved by the Experimental Animal Ethics Committee of Kyorin University School of Medicine, Tokyo, and all experimental animals were maintained in the animal facility in a specific-pathogen-free unit with sterile bedding, food, and water. Female mice (9–10 weeks old) were mated for 1 day with a male B6 mouse aged > 9 weeks and examined for the presence of a vaginal plug the next morning. Mice with or without a vaginal plug were infected with malaria parasites on day 12 post-mating.

### DNA constructs

The SK-1 construct contained a selection cassette consisting of green fluorescent protein gene (*gfp*) and a pyrimethamine resistance gene, human dihydrofolate reductase-thymidylate synthase (*hdhfr*) [33]. The expression of *gfp* and *hdhfr* is controlled by *hsp70* (PBANKA_071190) and *elongation factor-1* (PBANKA_113340) promoters, respectively. Plasmid containing luciferase gene (pLG4.10[*luc2*]) was purchased from Promega (Madison, WI, USA). To generate the luciferase-expressing cassette, luciferase gene (*luc2*) in the plasmid was amplified by PCR using specific primers (S1 Table). The PCR product of *luc2* was cleaved using the NheI and BglII restriction enzymes, and *gfp* of SK-1 was replaced with *luc2*. Luciferase-expressing cassette was introduced into the ORF of the targeted gene by double-crossover homologous recombination [34]. The gene-targeting vector was prepared by PCR [35]. Briefly, the 5′ and 3′ regions flanking the ORF of the target genes, *p230* [36], were amplified by PCR. The PCR products were annealed to either side of the luciferase-expressing cassette and amplified by PCR using gene-specific primers (S1 Table).

### Parasites and infections

*Pb* NK65 is a lethal strain and was originally obtained from Dr. M. Yoeli (New York University Medical Center, New York, NY, USA). Infected erythrocytes of *Pb* NK65 parasites were cultured for 18 h under standardized *in vitro* culture conditions. Mature schizonts were then collected by Nycodenz density-gradient centrifugation [34]. Transformations were performed using the Amaxa Basic Parasite Nucleofector Kit (Amaxa GmbH, Cologne, Germany) according to the manufacturer’s protocol. Briefly, 5 × 10^6^ to 5 × 10^7^ purified *Pb* NK65 mature schizonts were mixed with 100 μL of Nucleofector solution containing 5 μg of gene-targeting vector. Transfections were then completed using the Amaxa Nucleofector electroporation program U-33. Transfected parasites were injected intravenously (i.v.) into naïve B6 recipient mice. At 30 h post-injection, transfected parasites were isolated by the addition of pyrimethamine to the drinking water of infected mice. After parasitemia returned to detectable levels post-selection, transfected parasites were cloned by limiting dilution, after which a single parasite was injected into a mouse to ensure a clonally pure population. Cloned transfected parasites were stored as frozen stocks in liquid nitrogen. Infected erythrocytes of transfected parasites were generated in donor mice inoculated intraperitoneally with each frozen stock of parasite. The donor mice were monitored for parasitemia daily and bled for experimental infection in ascending periods of parasitemia. Experimental mice were infected intravenously with 1 × 10^4^ infected erythrocytes or 5 × 10^6^ to 5 × 10^7^ purified mature schizonts of a given parasite strain.

### Parasitemia and hematocrit

Blood was observed by microscopic examination of methanol-fixed tail blood smears stained with 3% Giemsa diluted with phosphate buffer, pH 7.2, for 45 min. The number of infected erythrocytes in 250 erythrocytes was enumerated when parasitemia exceeded 10%, whereas 1 × 10^4^ erythrocytes were examined when mice showed lower parasitemia. The percentage of parasitemia was calculated as follows: [(number of infected erythrocytes)/(total number of erythrocytes)] × 100.

For hematocrit measurement, blood was obtained from pregnant uninfected and pregnant infected mice on days 5 and 7 post-infection. The blood (50 μL) was collected into a heparinized capillary tube and centrifuged at 12,000 × *g* for 5 min with a micro-hematocrit centrifuge (HC–12A; Tomy, Tokyo, Japan). Hematocrit was expressed as the percentage of blood cells in the total volume of blood.

### *Ex vivo* organ bioluminescent imaging

Bioluminescent imaging was performed with a Photon IMAGER system (Biospace Lab, Nesles la Vallée, France). Mice were anesthetized and administered 1 mg of VivoGlo™ Luciferin (*In Vivo* Grade) dissolved in 150 μL of phosphate buffered saline (PBS) by i.v. injection. After receiving the VivoGlo™ Luciferin, mice were killed and organs were collected for image acquisition. Acquisition of emitted photons, with a charge-coupled device camera, was monitored. *Ex vivo* bioluminescent imaging data were analyzed using the M3software (Biospace) with size-constant regions of interest (ROIs).

### Enzyme–linked immunosorbent assay (ELISA) and antibodies

Blood was centrifuged at 500 × *g* for 10 min. The resulting supernatants were stored at –20°C and used as plasma. An ELISA for the detection of IFN-γ or IL-10 in plasma was performed as described previously [37, 38]. A rat anti-mouse IFN-γ mAb (clone R4–6A2; eBioscience) and a rat anti-mouse IL-10 mAb (clone JES5–16E3; eBioscience) were used as capture antibodies. A biotinylated, rat anti-mouse IFN-γ mAb (clone XMG1.2; eBioscience) and rat anti-mouse IL-10 mAb (clone JES5–2A5; eBioscience) were used as the detecting antibodies. The reaction was visualized by peroxidase-conjugated streptavidin (Zymed) and the substrate, 2,2’-azino-bis (3-etylbenzthiazoline-6-sulfonic acid) (ABTS) (Wako, Osaka, Japan). The absorbance of individual wells was determined using a Multiskan FC microplate reader (Thermo Fisher Scientific Inc., Waltham, MA, USA) at a wavelength of 414 nm. The concentrations of cytokines in plasma were calculated from standard curves prepared using known quantities of murine recombinant IFN-γ (Genzyme, Boston, MA, USA) and IL-10 (Pierce, Rockford, IL, USA). Purified antibodies for *in vivo* CD8^+^ cell-depletion (500 μg of anti-mouse CD8 mAb, clone 2.43, eBioscience) and neutrophil/macrophage-depletion (300 μg of anti-mouse Gr1 mAb, clone RB6-8C5; eBioscience, San Diego, CA, USA) were injected intraperitoneally into the mice.

### Histological examination of placentas

Placentas were obtained from uninfected pregnant mice and infected pregnant mice on day 6 post-infection (p.i.). Mice were killed and placentas were removed. The placentas were fixed in 10% buffered formalin and embedded in paraffin. Six-micrometer-thick sections were stained with hematoxylin and eosin (H&E). The stained thick sections were photographed at 20×, 100×, and 400× magnification using an All-in-One Fluorescence Microscope (BZ9000; KEYENCE Japan, Osaka, Japan).

### Flow cytometry

Placentas were removed from uninfected and infected mice on day 18 post mating. Mice were euthanized before removing the placentas. Flow cytometry analyses were performed using single-cell suspensions of the placenta, as described previously [37, 38]. The following monoclonal antibodies (mAbs) were used: FITC-conjugated anti-CD11b mAb (clone M1/70; eBioscience, San Diego, CA, USA) and anti-CD4 mAb (clone GK1.5; eBioscience); PE-conjugated anti-CD8 mAb (clone 53.6.7; eBioscience); and allophycocyanin-conjugated anti-CD3ε mAb (clone 145–2C11; eBioscience) and anti-F4/80 mAb (clone BM8; eBioscience). MAbs were added to cells in FACS buffer (1% BSA, 0.1% sodium azide in PBS), incubated at 4°C for 30 min, washed with cold FACS buffer, and then centrifuged at 250 × *g* for 2 min. Then they were incubated at 4°C for 30 min, washed with cold FACS buffer, and centrifuged at 250 × *g* for 2 min before being fixed with 1% paraformaldehyde. Flow cytometry was performed with FACSCalibur (BD Biosciences, San Jose, CA, USA) and analyzed using FlowJo.

### Statistical analysis

Parasitemia, hematocrit, luciferase activity, and levels of cytokines were analyzed using the Student’s t-test, which was performed using Statcel (OMS, Saitama, Japan). *P* values of less than 0.05 were considered statistically significant.

## Results

### Pregnant mice show severe pathology and adverse pregnancy outcomes during infection with malaria parasites

**Fig 1:**
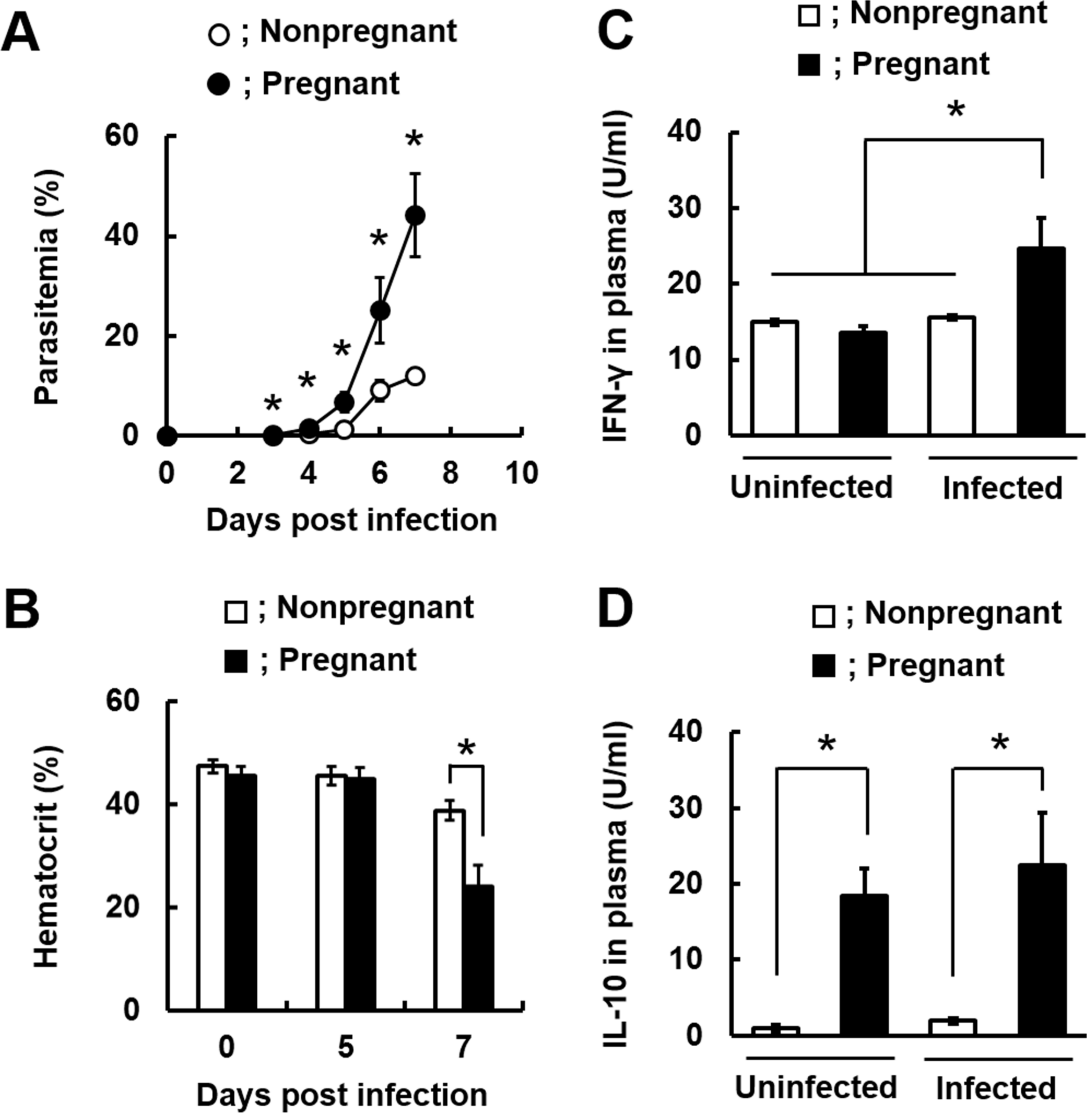
The increase in susceptibility of mice to infection with *Pb*NK65L during pregnancy. Female C57BL/6 (B6) mice on day 12 post-mating were infected with 1 × 10^4^ infected erythrocytes of luciferase-expressing *P*. *berghei* NK65 (*Pb*NK65L). (A) Course of parasitemia in nonpregnant and pregnant mice infected with *Pb*NK65L. (B) Hematocrit on days 0, 5, and 7 post-infection (p.i). (C) Plasma IFN-γ levels on day 5 p.i. (D) Plasma IL-10 levels on day 5 p.i. Asterisks indicate a statistically significant difference (*p* < 0.05). Results are expressed as means ± SD of three to five mice. Experiments were performed in duplicate with similar results.

We first generated luciferase-expressing *Plasmodium berghei* NK65 (*Pb*NK65L) (S1 Fig) and examined the outcomes of infection with *Pb*NK65L. As shown in Fig 1A, parasitemia of pregnant mice infected with *Pb*NK65L was rapidly increased compared with nonpregnant mice from day 3 p.i. In pregnant mice infected with *Pb*NK65L, the levels of hematocrit were comparable to those in infected nonpregnant mice until day 5 p.i. (Fig 1B). However, infected pregnant mice showed lower levels of hematocrit than those in infected nonpregnant mice on day 7 p.i. (Fig 1B). Preterm delivery was observed in pregnant mice infected with *Pb*NK65L and the survival rate of pups was decreased compared with that of uninfected mice (Table 1). These results suggest that pregnant mice show severe pathology compared with nonpregnant mice and adverse pregnancy outcomes during infection with malaria parasites.

**Table 1:** The pregnancy outcomes in pregnant mice infected with *Pb*NK65L.

Mice^a^	Parasites	Number of pup	Number of live-pup	Survival rate of pup (%)	Pregnancy period (day)			
**Wild type**	**Uninfected**	**21**	**21**	**100**	**20.00**	**±**	**0.00**	
**Wild type**	***Pb*NK65L**	**18**	**1**	**5.56**	**18.00**	**±**	**0.00**	***p* < 0.05**
**IFN-γR1-KO**	***Pb*NK65L**	**23**	**18**	**78.26**	**19.67**	**±**	**0.58**	
**CD8-depleted**^**b**^	**PbNK65L**	**17**	**0**	**0**	**17.67**	**±**	**0.58**	**p < 0.05**
**Gr-1-depleted**^**c**^	***Pb*NK65L**	**22**	**0**	**0**	**17.00**	**±**	**0.00**	***p* < 0.05**

a; Mice on day 12 post-mating were infected with 1 × 10^4^ erythrocytes infected with luciferase-expressing *P*. *berghei* NK65 (*Pb*NK65L).

b; Anti-CD8 mAbs were injected into infected WT mice on day 3 p.i.

c; Anti-Gr-1 mAbs were injected into infected WT mice on day 4 p.i.

Experiments using three mice were performed in duplicate with similar results. *P* values indicate significant differences compared to the pregnancy period of uninfected mice.

To examine whether preterm delivery and fetal death occur during infection with malaria parasites, pregnant mice on day 12 or 15 post-mating were infected with intact *Pb*NK65L (S2 Table). In pregnant mice on day 12 post-mating, preterm delivery and fetal death were observed during infection (S2 Table). In contrast, pregnant mice on day 15 post-mating successfully delivered live pups during infection (S2 Table). Since the pregnancy period was 18 days in mice that were infected with *Pb*NK65L on day 12 post-mating, the risk of preterm delivery appears to increase from day 6 p.i. Several studies reported that production of pro-inflammatory cytokines, such as IFN-γ and TNF-α, was enhanced in placenta from pregnant women or mice infected with malaria parasites [15–19, 30, 31]. To examine whether the response of pro-inflammatory cytokines is involved in adverse pregnancy outcomes, we first measured levels of cytokines in plasma on day 5 p.i. The high levels of IFN-γ were observed in infected pregnant mice compared with those in infected nonpregnant mice on day 5 p.i. (Fig 1C). Analyses of cytokines in plasma detected high levels of IL-10 in uninfected and infected pregnant mice on day 5 p.i. (Fig 1D). These findings suggest that the immune response activated by IFN-γ plays an important role in adverse pregnancy outcomes during *Pb*NK65L infection.

### Pregnant IFNGR1-KO mice infected with malaria parasites successfully delivered live pups

**Fig 2:**
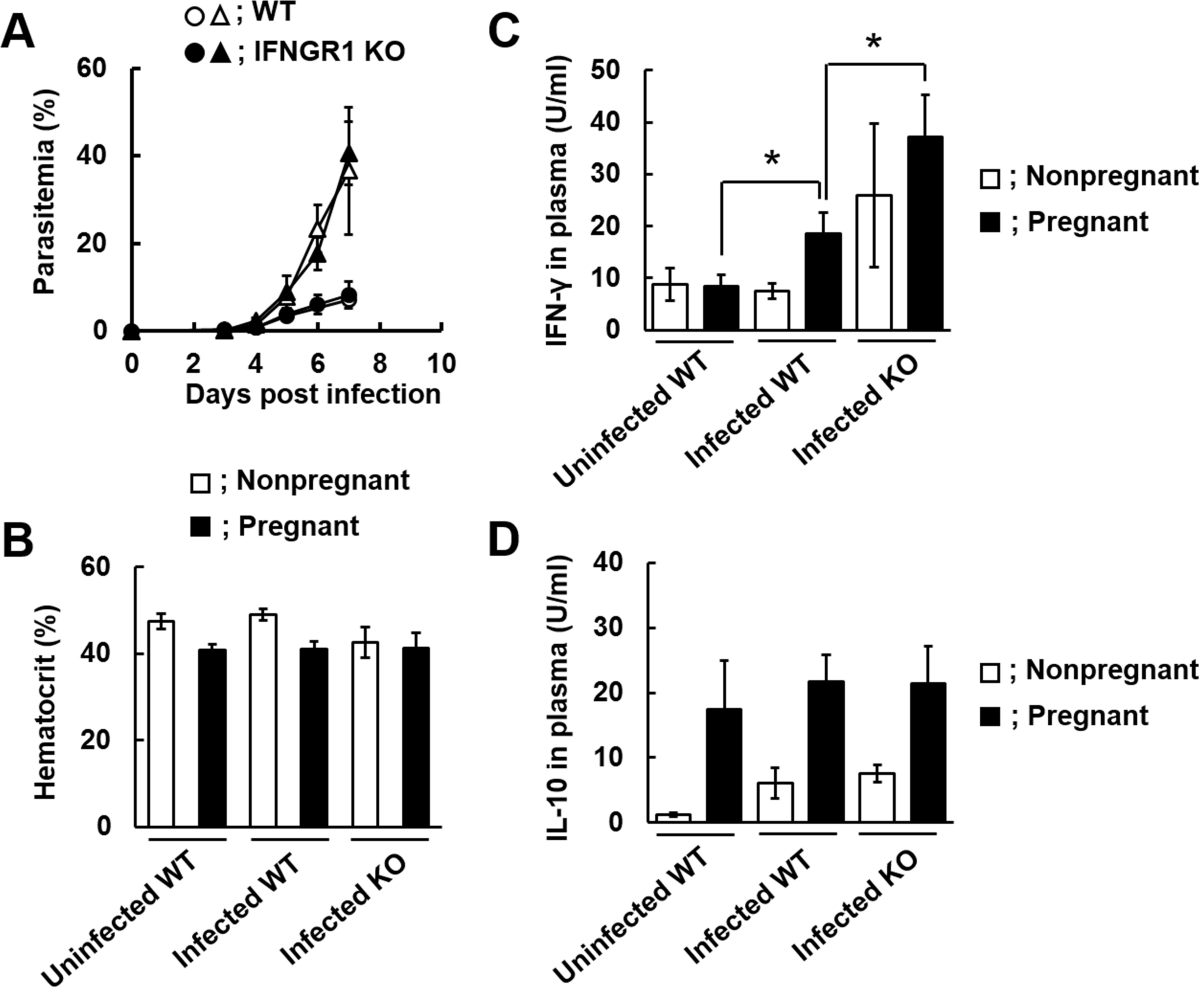
The effect of IFNGR1 deficiency on the outcome of infection with *Pb*NK65L during pregnancy. Female wild type (WT) and IFN-γ receptor 1-deficient (IFNGR1-KO) mice were put together with male WT mice for 1 day. Mice on day 12 post-mating were infected with 1 × 10^4^ infected erythrocytes of *Pb*NK65L. (A) Course of parasitemia in WT and IFNGR1-KO mice. Parasitemia was observed in nonpregnant (circles) and pregnant (triangles) mice infected with *Pb*NK65L. (B-D) Blood or plasma were obtained from uninfected wild type mice (Uninfected WT), WT mice infected with *Pb*NK65L (Infected WT), or IFNGR1-KO mice infected with *Pb*NK65L (Infected KO) mice on day 6 p.i. (B) Hematocrit in nonpregnant and pregnant mice on day 6 p.i. (C) Plasma IFN-γ levels in nonpregnant and pregnant mice on day 6 p.i. (D) Plasma IL-10 levels in nonpregnant and pregnant mice on day 6 p.i. Asterisks indicate a significant difference (*p* < 0.05). Results are expressed as means ± SD of three mice. Experiments were performed in triplicate with similar results.

To investigate whether the immune response to IFN-γ is involved in adverse pregnancy outcomes during malaria infection, pregnant IFN-γ receptor 1-deficient (IFNGR1-KO) mice were infected with *Pb*NK65L. As shown in Fig 2A, parasitemia in pregnant and nonpregnant mice infected with *Pb*NK65L was not affected by deficiency of IFNGR1. However, the survival rate of pups of pregnant IFNGR1-KO mice was higher than that of wild-type (WT) mice during infection (Table 1). Preterm delivery, which was observed in infected pregnant WT mice on day 6 p.i., did not occur in pregnant IFNGR1-KO mice (Table 1). Therefore, we next examined the hematocrit and IFN-γ and IL-10 levels in pregnant WT and IFNGR1-KO mice on day 6 p.i. In pregnant IFNGR1-KO mice infected with *Pb*NK65L, the hematocrit was comparable to that in infected pregnant WT mice on day 6 p.i. (Fig 2B). High IFN-γ levels were observed in infected pregnant IFNGR1-KO mice compared to those in infected pregnant WT mice on day 6 p.i. (Fig 2C), while the IL-10 levels in infected pregnant IFNGR1-KO mice were comparable to those in infected pregnant WT mice on day 6 p.i. (Fig 2D).

### Erythrocytes infected with *Pb*NK65L accumulate in the lung, spleen, adipose tissue, and placenta in pregnant IFNGR1-KO mice

**Fig 3:**
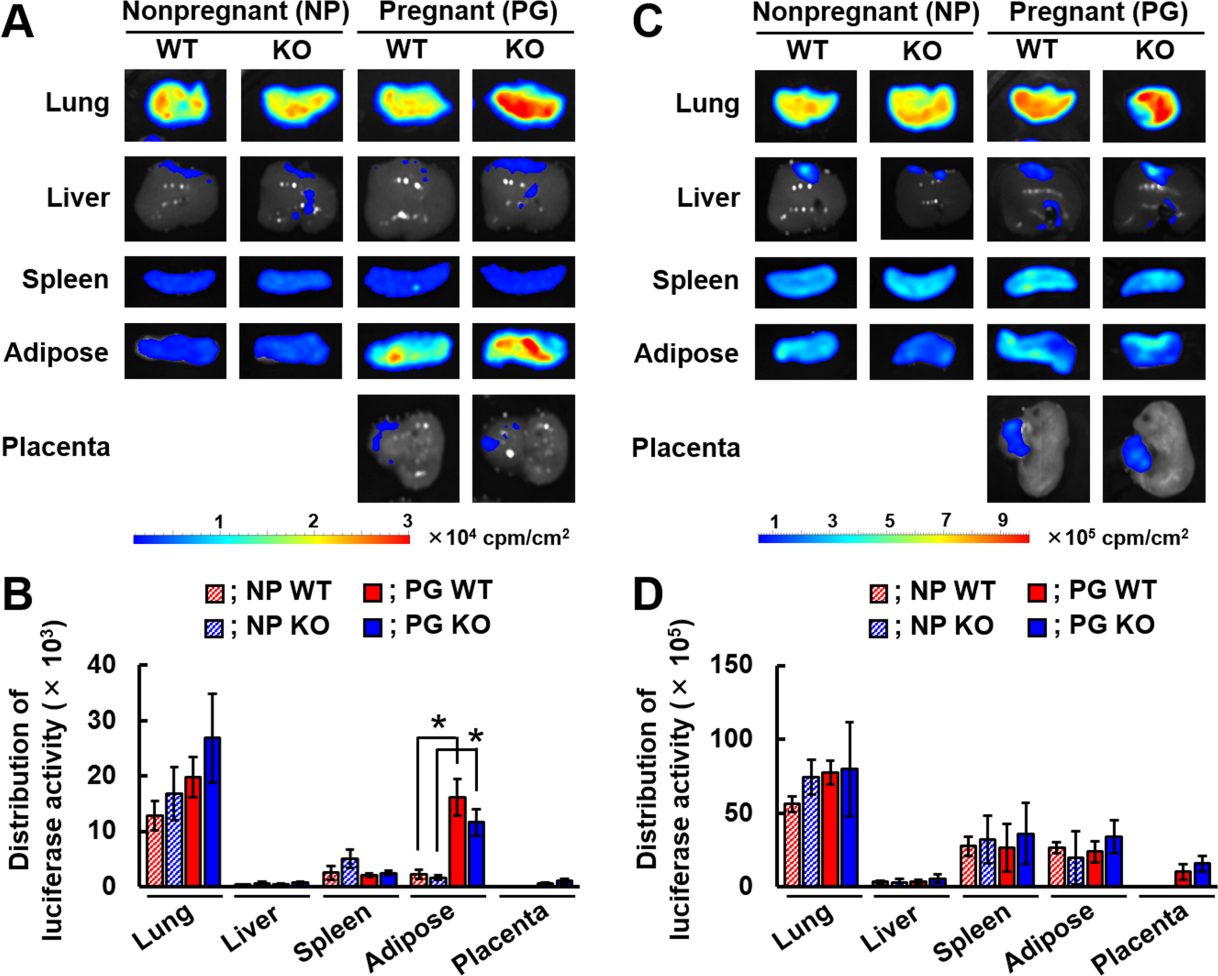
The effect of IFNGR1 deficiency on the localization of infected erythrocytes in pregnant mice. *Ex vivo* bioluminescent images of luciferase expression in organs of WT or IFNGR1-KO mice following infection with *Pb*NK65L. Nonpregnant (NP) and pregnant mice (PG) on day 12 post-mating were infected with 1 × 10^4^ infected erythrocytes of *Pb*NK65L. D-luciferin (50 mg) was injected into the tail vein of NP and PG mice and the organs of mice from each group were removed on day 3 p.i. (A and B) or day 6 p.i. (C and D). (A and C) Bioluminescent images of luciferase activity in the organs of mice from each group. Representative data are shown. (B and D) The bioluminescent signal from each organ was quantified using the Living Image software. Asterisks indicate a statistically significant difference (*p* < 0.05). Results are expressed as means ± SD of three mice. Experiments were performed in duplicate with similar results.

Next, we investigated the organs in which *Pb*NK65L-infected erythrocytes accumulate in pregnant IFNGR1-KO mice using bioluminescence imaging. On day 3 p.i., luciferase activities were detected in the lung, liver, spleen, subcutaneous adipose tissue, and placenta of infected pregnant WT and IFNGR1-KO mice (Fig 3A). However, the luciferase activities in the liver and placenta were much lower than those in the lung, spleen, and subcutaneous adipose tissue (Fig 3A). By contrast, in infected pregnant WT and IFNGR1-KO mice, the luciferase activity in subcutaneous adipose tissue was higher than in infected nonpregnant WT and IFNGR1-KO mice (Fig 3A and 3B). On day 6 p.i., high luciferase activity was detected in the lung, spleen, subcutaneous adipose tissue, and placenta in infected pregnant WT and IFNGR1-KO mice (Fig 3C), while the distribution of luciferase activity in organs from infected pregnant IFNGR1-KO mice was similar to that in infected pregnant WT mice on day 6 p.i. (Fig 3C and 3D). These results suggest that the accumulation of erythrocytes infected with *Pb*NK65L was not affected by IFNGR1 deficiency.

### IFNGR1 signaling plays a crucial role in placental inflammation during *Pb*NK65L infection

**Fig 4:**
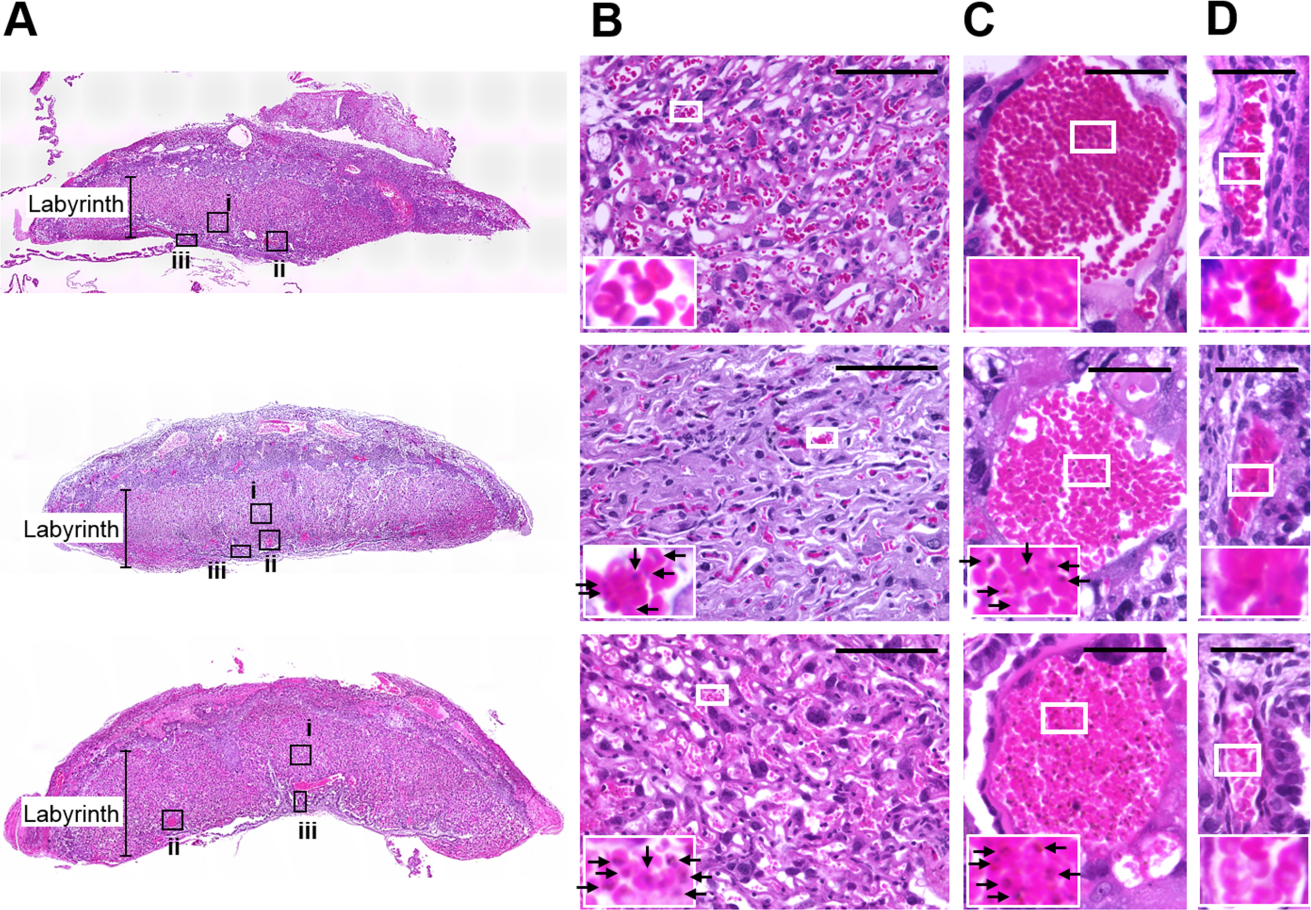
Role of IFNGR1 signaling in placental inflammation during *Pb*NK65L infection. Representative hematoxylin and eosin (H&E)-stained placental sections are shown. Placentas were obtained from uninfected pregnant mice (Uninfected WT, top panels), pregnant WT mice infected with *Pb*NK65L (infected WT, middle panels), or pregnant IFNGR1-KO mice infected with *Pb*NK65L (Infected KO, bottom panels) mice on day 6 p.i. (A) Low magnification images of placenta. (B) Higher magnification of labyrinth region (black boxes (i) in A). The scale bars indicate 100 μm. (C) Higher magnification of maternal blood vessel (black boxes (ii) in A). The scale bars indicate 40 μm. (D) Higher magnification of fetal blood vessel (black boxes (iii) in A). The scale bars indicate 40 μm. Regions indicated by white boxes in B–D were enlarged to inset boxes. Arrows indicate *Pb*NK65L-infected erythrocytes. Experiments using three to five mice were performed in triplicate with similar results, and representative data are shown.

We then performed histological analyses of placenta (Fig 4). As a result, a decreasing number of vascular branches and accumulation of infected erythrocytes were observed in the labyrinth of placentas from infected pregnant WT mice, but not in uninfected pregnant WT mice (Fig 4A and 4B). However, accumulation of infected erythrocytes within fetal vascular were not observed in the placentas from infected pregnant WT mice (Fig 4C and 4D). In contrast, the decreasing numbers of vascular branches were improved in the labyrinth of placentas from infected pregnant IFNGR1-KO mice (Fig 4A and 4B). On the other hand, accumulation of infected erythrocytes within maternal vasculature, but not within fetal vasculature, was observed (Fig 4C and 4D). These results suggested that IFNGR1 signaling is involved in placental inflammation during *Pb*NK65L infection.

### Accumulation of CD8^+^ T cells and F4/80^+^ cells in the placenta is suppressed in IFNGR1-KO mice

**Fig 5:**
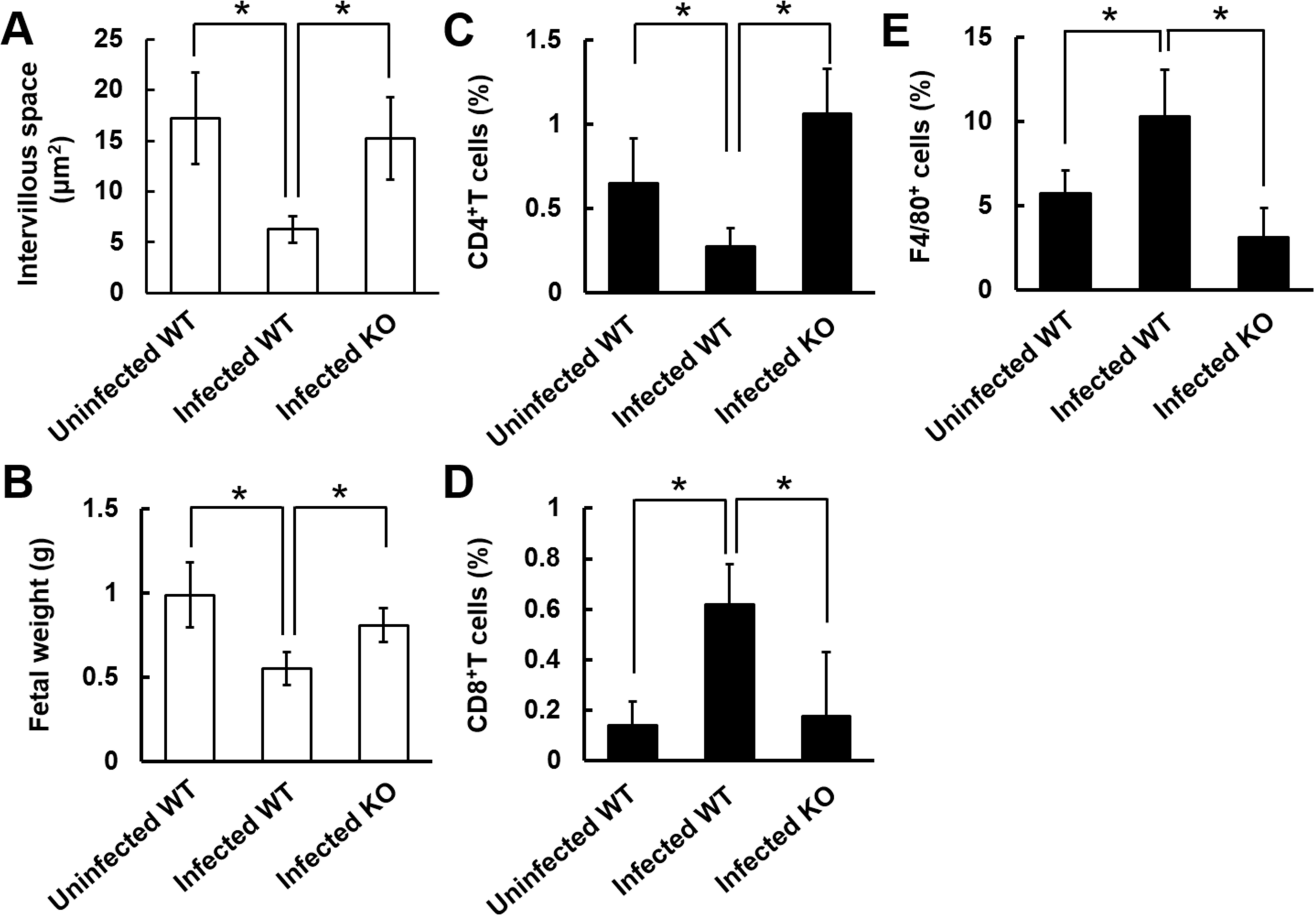
CD8^+^ T cells and F4/80^+^ cells are involved in the development of placental pathology during malaria. (A) Intervillous space in the labyrinth of placentas on day 6 p.i. The labyrinth region of the intervillous space is shown at higher magnification in Fig 4B. (B) Fetal weight on day 6 p.i. (C) The proportion of CD4^+^ cells in the CD3^+^ gate in placentas on day 6 p.i. (D) The proportion of CD8^+^ cells in the CD3^+^ gate in placentas on day 6 p.i. (E) The proportion of F4/80^+^ cells in the CD11b^+^ gate in placentas on day 6 p.i. Asterisks indicate a significant difference (*p* < 0.05). Results are expressed as means ± SD of three mice. Experiments were performed in duplicate with similar results.

During pregnancy, in the labyrinths of placentas from infected WT mice, the intervillous space and fetal weight were significantly decreased compared with those in uninfected WT mice on day 6 infection (Fig 5A and 5B). The decreased intervillous space and fetal weight were improved in infected IFNGR1-KO mice (Fig 5A and 5B). To investigate the effect of IFNGR1-deficiency on the immune response in the placenta during malaria infection, CD4^+^T, CD8^+^T, and F4/80^+^ cells in the placenta were assessed by flow cytometry (Fig 5C–5E and S2 Fig). The proportion of CD4^+^T cells in placentas from infected WT mice was lower than that in uninfected mice (Fig 5C), while the proportions of CD8^+^T and F4/80^+^ cells in placentas from infected WT mice were higher than those in uninfected mice (Fig 5D and 5E). The decreased proportion of CD4^+^T cells and increased proportions of CD8^+^T and F4/80^+^ cells were recovered in IFNGR1-deficiency (Fig 5C–5E). These findings suggest that IFNGR1 signaling is involved in the increases in CD8^+^T and F4/80^+^ cells in the placenta. Therefore, we examined the effect of CD8^+^T cell and neutrophil/macrophage depletion on pregnancy outcomes during malaria infection. We observed adverse pregnancy outcomes during malaria infection in CD8^*+*^T cell-depleted or neutrophil/macrophage-depleted mice (Table 1). Our findings suggest that CD8^+^T and F4/80^+^ cells might not be associated with the development of placental pathology during malaria.

## Discussion

This study explored the role of IFNGR1 in the pathogenesis of placental pathology using a mouse model of malaria during pregnancy. The bioluminescence imaging results suggested that the accumulation of *Pb*NK65L-infected erythrocytes in organs such as the lung, spleen, subcutaneous adipose tissue, and placenta in infected pregnant IFNGR1-KO mice was comparable to that in infected pregnant WT mice. The *Pb*NK65L-infected pregnant WT mice showed adverse pregnancy outcomes. Here, we found that fetal mortality in *Pb*NK65L-infected IFNGR1-KO mother mice was significantly lower than in WT mice infected with *Pb*NK65L. Histological analyses of placentas showed that damage to the chorionic villi in *Pb*NK65L-infected IFNGR1KO mice was improved compared with that in infected WT mice. These findings suggest that the accumulation of infected erythrocyte to placenta could induce inflammatory responses via IFNGR1 signaling, and are associated with adverse pregnancy outcomes during infection with malaria parasites.

Numerous studies have shown that placental inflammation is induced in women and mice infected with malaria parasites [16–18]. Malaria parasites are recognized by pathogen-associated molecular patterns, such as toll-like receptors (TLRs), of host immune cells [39]. In addition to immune cells, the expression of the TLR family was observed in trophoblasts and syncytiotrophoblasts [40, 41]. In a mouse model, *P*. *berghei* NK65-infected erythrocytes adhered to placental tissue by binding to CSA and were involved in the induction of pro-inflammatory cytokines in placental tissue [30]. High levels of pro-inflammatory cytokines in the placenta have been shown to be abrogated by deficiency in MyD88 during malarial infection [30], considering that pro-inflammatory cytokines such as IFN-γ may be produced by host immune cells and trophoblasts when TLR signaling is activated by the accumulation of infected erythrocytes in the placenta.

In this study, the parasite accumulation in the placentas of pregnant IFNGR1-deficient mice was comparable to that in WT mice infected with *Pb*NK65L. However, the proportions of CD8^+^T and F4/80^+^ cells in the placentas of infected IFNGR1-deficient mice were lower than those in infected WT mice. These results suggest that IFNGR1 signaling is involved in the increase in immune cells, such as CD8^+^ cells, neutrophils, and macrophages, in placental tissue. IFN-γ is associated with fetal abortion and resorption during *Plasmodium* and bacterial infection [19, 42]. The infiltration of neutrophils and macrophages is observed in placentas from patients infected with malaria parasites [13, 14]. During bacterial infection, CD8^+^T cells and neutrophils are related to adverse pregnancy outcomes [42]. Although mice were depleted of CD8^+^ cells or neutrophil/macrophages, preterm delivery and the pup survival rate were not improved in CD8^+^ cell- or neutrophil/macrophage-depleted mice (Table 1). These findings suggest that both CD8^+^T cells and F4/80^+^ cells might not be involved in the development of placental pathology and the mechanism by which placental pathology is developed during *Plasmodium* infection may be more complicated than in bacterial infection.

TNF-α is one of the major macrophage-produced cytokines and is associated with the development of placental pathology during malaria [31]. Although IFN-γ is a major inducer of TNF production, Poovassery et al. [31] have shown that pregnant IFN-γ deficient mice infected with *P*. *chabaudi* AS exhibited high levels of TNF compared to uninfected pregnant mice. For instanse, high levels of TNF-α activity were detected when monocytes were cocultured with *P*. *falciparum* schizont stage-parasitized erythrocytes or pigment recovered from ruptured schizonts [43, 44]. The previous study by Poovassery et al. [31] suggest that IFNGR1 signaling may affect the expression level of TNF receptor or number of TNF receptor expressed cells but not affect TNF production during malaria. IFN-γ production is restricted to cells of the immune system [45]. However, because IFNGR1/2 proteins are widely expressed, nearly all cell types are capable of responding to IFN-γ [45]. Moreover, it is reported that IFNGR1 is also localized in the placenta throughout pregnancy [46, 47]. It remains unclear which cells are important for IFN-γ signaling in the development of placental pathology during malaria.

We observed high levels of luciferase activity in adipose tissue in infected pregnant mice on day 3 p.i. A study using *P*. *berghei* ANKA showed that schizont membrane-associated cytoadherence protein (SMAC) is involved in the CD36-mediated sequestration of schizonts in adipose tissue [48]. The accumulation of erythrocytes infected with *Pb*NK65L in subcutaneous adipose tissue in pregnant mice may be involved in the CD36-mediated sequestration via SMAC. During pregnancy, lipid metabolism is drastically changed for fetal development through the action of pregnancy hormones, such as progesterone [49] or human chorionic gonadotropin [50]. Therefore, the accumulation of infected erythrocytes into adipose tissue may be due to enhanced expression of CD36 on adipose tissue or increases in CD36-expressing adipocytes during pregnancy. Our findings suggest that the accumulation of infected erythrocytes in adipose tissue increases the sensitivity and specificity of detecting infection during pregnancy.

A rapid increase of parasitemia in pregnant mice was observed compared with that in nonpregnant mice. Regulatory T cells have been shown to increase during pregnancy [51]. Therefore, it is believed that the increase in susceptibility of mice to infection with malaria parasites may be associated with regulatory T cells during pregnancy. Pregnancy-associated hormones, such as progesterone (P4) and 17β-estradiol (E2), are known to enhance the suppressive capacity of regulatory T cells [52]. Although no effect of treatment of P4 and/or E2 on the course of parasitemia was observed in mice infected with *P*. *chabaudi*, treatment with E2 increased levels of IL-10 in the infected mice [53]. In fact, high levels of IL-10 were observed in pregnant mice in this study. On the other hand, maternal metabolism, such as glucose, lipid [54, 55], and purine metabolism [56], changes significantly during pregnancy considering that the alteration in host nutrient conditions may be associated with an enhancement of parasite-growth during pregnancy.

Our finding that IFNGR1 signaling plays a central role in the development of placental pathology during malaria in pregnancy suggests that blocking IFNGR1 signaling may be effective for the prevention of adverse pregnancy outcomes during infection with malaria parasites. In addition, our findings suggest that the accumulation of infected erythrocytes in adipose tissue in pregnant mice during the early phase of infection may contribute to the identification of a parasite marker for early detection of malaria parasites during pregnancy. Based on these preclinical findings, additional investigations are required to establish whether maternal IFNGR1 signaling is also involved in developing placental pathology in clinical cases of severe malaria.

## Supporting information

S1 Fig Generation of luciferase-expressing *Plasmodium berghei* NK65. Schematic representation of gene-targeting vectors (A and B). SK-1 vector (A) and Sk-1-luc2 vector (B). Restriction sites of NheI and BglII restriction enzymes were shown. (C) Luciferase (*luc2*)-expressing cassette was introduced into target gene by double-crossover homologous recombination. Arrows (F1 and F2) denote primers specific for the 5′ and 3′ regions of the target gene (S1 Table). Introduction of *luc2* into the *p230* locus (PBANKA_030600), which is not essential in the complete life cycle of the parasite [36], of *Pb*NK65 parasites. Proper integration was confirmed using primers specific for *p230* (WT, 4.7 kbp; Introduced, 7.0 kbp) for two cloned transfected parasites. Parasites in which the *luc2*-expressing cassette was introduced into the *p230* locus were used as control *Pb*NK65L in this study.
(TIF)

S2 Fig Assessment of CD4+T, CD8+T, and F4/80+ cells in the placenta by flow cytometry. Placenta were obtained from uninfected wild type mice (Uninfected WT), WT mice infected with *Pb*NK65L (Infected WT), or IFNGR1-KO mice infected with *Pb*NK65L (Infected KO) mice on day 6 p.i. (A) The dot plots of CD4^+^ cells and CD8^+^ cells in the CD3^+^ gate in placentas on day 6 p.i. (B) The histograms of F4/80^+^ cells in the CD11b^+^ gate in placentas on day 6 p.i.
(TIF)

S1 Table Sequence of primers used in this study. (XLSX)

S2 Table The effect of different timing of infection on the pregnancy outcomes. a; Mice on day 12, or 15 post-mating were infected with 1 × 10^4^ erythrocytes infected with luciferase-expressing *P*. *berghei* NK65 (*Pb*NK65L).
Experiments using three mice were performed in duplicate with similar results. *P* values indicate a significant difference compared to pregnancy period of uninfected mice.
(XLSX)
