# Supplementary figures and images for: IFNGR1 signaling is associated with adverse pregnancy outcomes during infection with malaria parasites
Source: PLoS One. 2017 Nov 8;12(11):e0185392. doi: 10.1371/journal.pone.0185392 (PMC5678718; doi:10.1371/journal.pone.0185392)

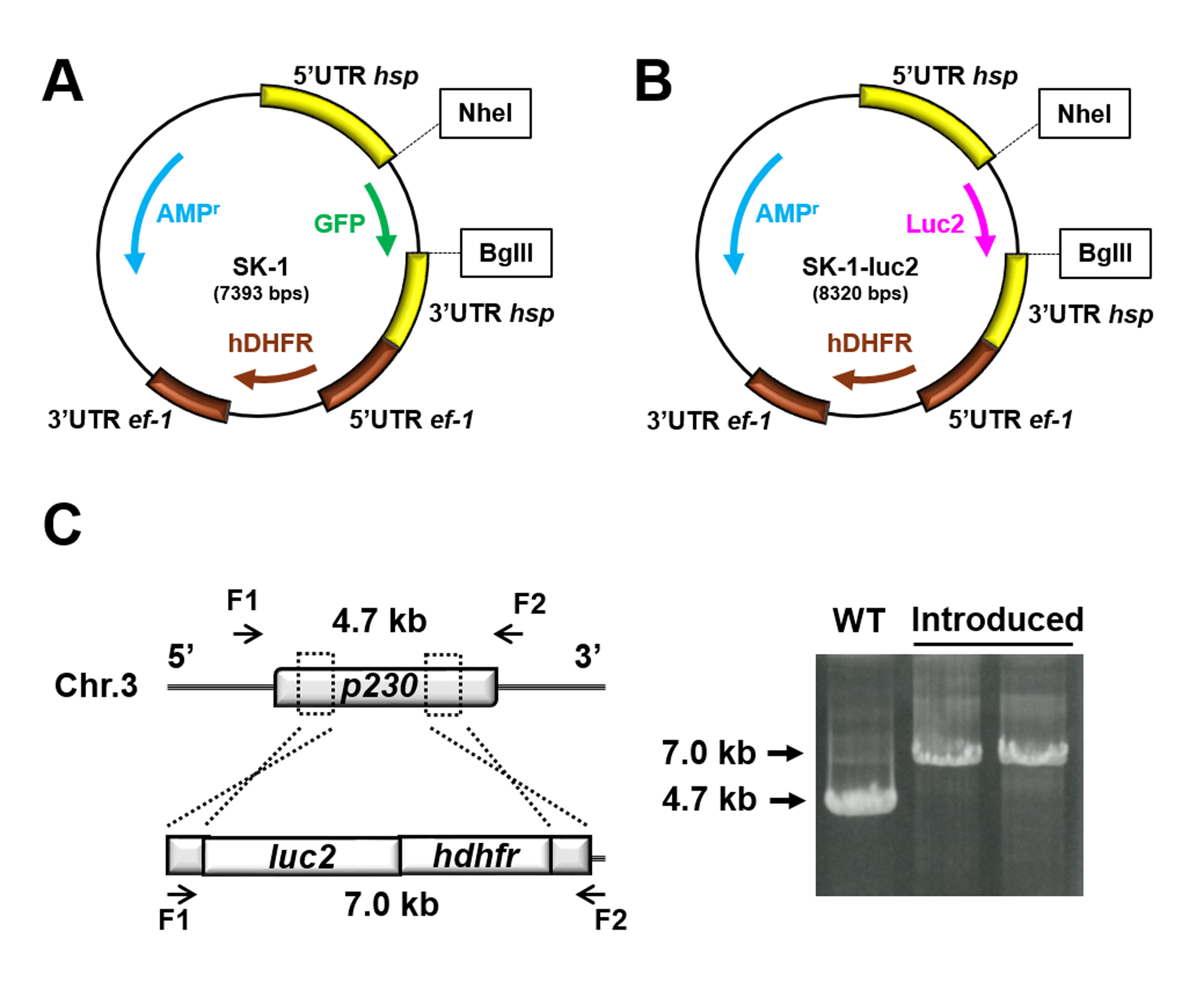

Supplement: S1 Fig — Schematic representation of gene-targeting vectors (A and B). SK-1 vector (A) and Sk-1-luc2 vector (B). Restriction sites of NheI and BglII restriction enzymes were shown. (C) Luciferase (luc2)-expressing cassette was introduced into target gene by double-crossover homologous recombination. Arrows (F1 and F2) denote primers specific for the 5′ and 3′ regions of the target gene (S1 Table). Introduction of luc2 into the p230 locus (PBANKA_030600), which is not essential in the complete life cycle of the parasite [36], of PbNK65 parasites. Proper integration was confirmed using primers specific for p230 (WT, 4.7 kbp; Introduced, 7.0 kbp) for two cloned transfected parasites. Parasites in which the luc2-expressing cassette was introduced into the p230 locus were used as control PbNK65L in this study. (TIF) [file pone.0185392.s001.tif]

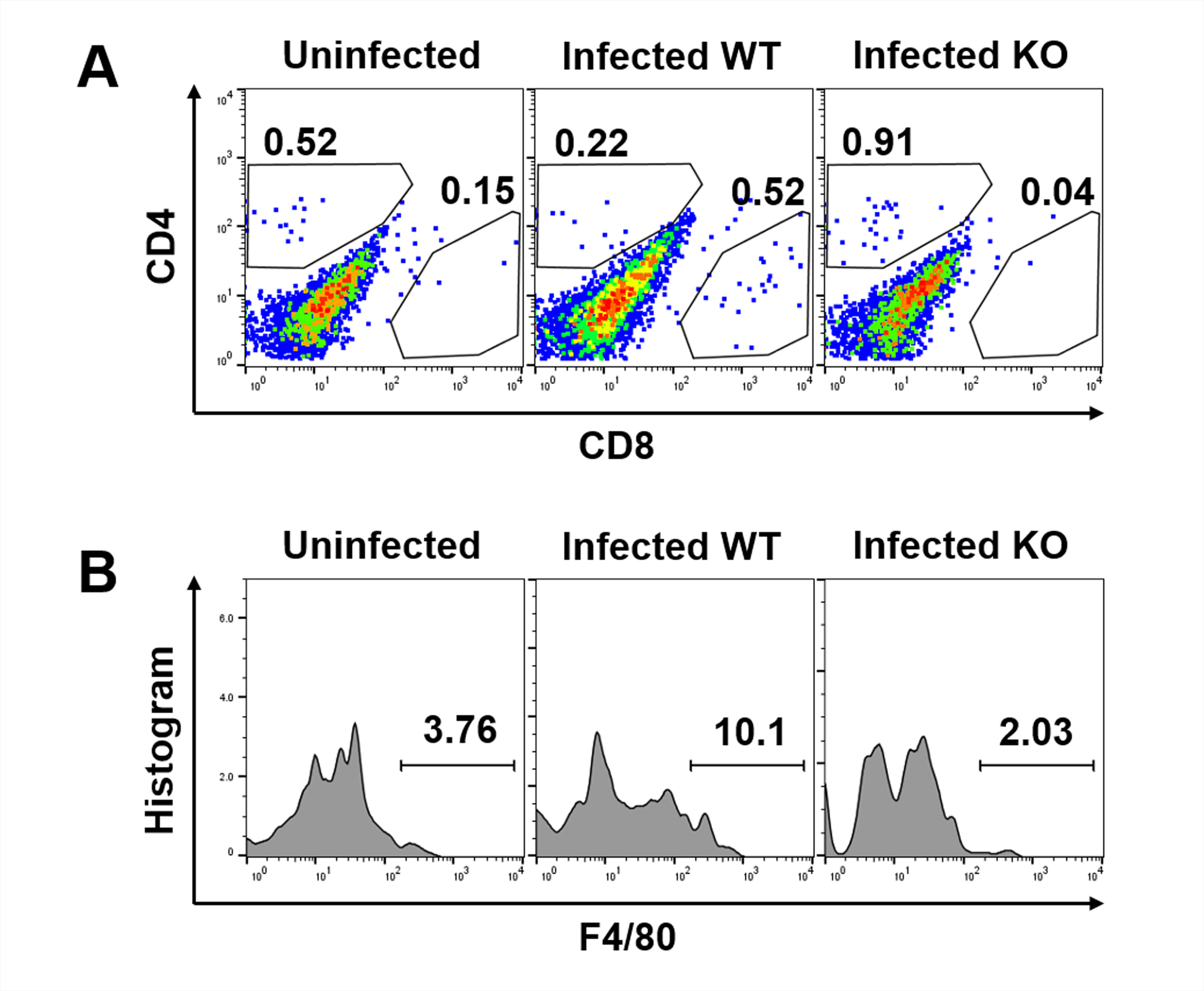

Supplement: S2 Fig — Placenta were obtained from uninfected wild type mice (Uninfected WT), WT mice infected with PbNK65L (Infected WT), or IFNGR1-KO mice infected with PbNK65L (Infected KO) mice on day 6 p.i. (A) The dot plots of CD4+ cells and CD8+ cells in the CD3+ gate in placentas on day 6 p.i. (B) The histograms of F4/80+ cells in the CD11b+ gate in placentas on day 6 p.i. (TIF) [file pone.0185392.s002.tif]
